# Correction: Secondary metabolites from *Lobaria pulmonaria* (L.) Hoffm. target key metabolic enzymes: a novel strategy against multidrug-resistant tuberculosis

**DOI:** 10.1039/d6ra90025a

**Published:** 2026-03-02

**Authors:** Ha Thi Nguyen, Vishnu Nayak Badavath, Siddhartha Maji, Haritha Polimati, Emmanuel Okello, Richard A. Bunce, Nguyen Huy Thuan, Wan Mohd Nuzul Hakimi Wan Salleh, Vinay Bharadwaj Tatipamula

**Affiliations:** a Center for Pharmaceutical Biotechnology, Duy Tan University Da Nang 550000 Vietnam; b Institute of Research and Development, Duy Tan University Da Nang 550000 Vietnam vinaybharadwajtatipamula@duytan.edu.vn; c School of Pharmacy and Technology Management, SVKM's Narsee Monjee Institute of Management Studies (NMIMS), Deemed-to-be-University Green Industrial Park TSIIC Jadcherla Hyderabad 509301 India; d Department of Chemistry, Oklahoma State University Stillwater 74078 Oklahoma USA; e Pharmacology Department, AU College of Pharmaceutical Sciences, Andhra University Visakhapatnam 530003 Andhra Pradesh India; f Veterinary Medicine Teaching and Research Center, School of Veterinary Medicine, University of California Davis Tulare CA USA; g Department of Chemistry, Faculty of Science and Mathematics, Universiti Pendidikan Sultan Idris Tanjong Malim 35900 Perak Malaysia

## Abstract

Correction for ‘Secondary metabolites from *Lobaria pulmonaria* (L.) Hoffm. target key metabolic enzymes: a novel strategy against multidrug-resistant tuberculosis’ by Ha Thi Nguyen *et al.*, *RSC Adv.*, 2026, **16**, 8960–8970, https://doi.org/10.1039/D5RA05774D.

The authors regret that several errors were introduced into the affiliations section of the published manuscript. The corrected affiliations are as shown here.

The Royal Society of Chemistry apologises for these errors and any consequent inconvenience to authors and readers.

